# Supplementary material for: Identification of photoperiod-induced specific miRNAs in the adrenal glands of Sunite sheep (Ovis aries)
Source: Front Vet Sci. 2022 Jul 22;9:888207. doi: 10.3389/fvets.2022.888207 (PMC9354845; doi:10.3389/fvets.2022.888207)
Supplement: Supplementary file 1 [file Data_Sheet_1.ZIP › Supplementary file/miRDeep2 file.zip!/miRDeep2 file/Novel_Structure/LP42A3/Novel_470.pdf]

Provisional ID	:	NC_019484.2_25332
Score total	:	59.8
Score for star read(s)	:	3.9
Score for read counts	:	52.6
Score for mfe	:	1.7
Score for randfold	:	1.6
Score for cons. seed	:	
Total read count	:	115
Mature read count	:	109
Loop read count	:	0
Star read count	:	6

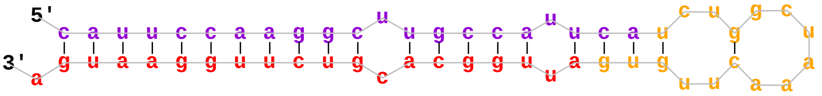

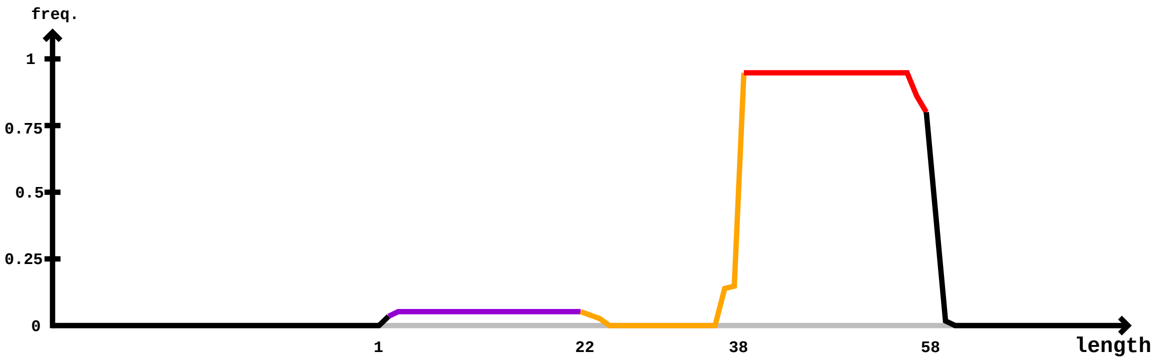

Star Mature

```
5'- uugccaccuucagcgugucugugugcuguaccccauuccaaggcuugccauucaucuggcuaaacuugugauuggcacgucuuggaaugaaguaacguacaacaaugaug  -3'   obs
    uugccaccuucagcgugucugugugcuguaccccauuccaaggcuugccauucaucuggcuaaacuugugauuggcacgucuuggaaugaaguaacguacaacaaugaug         exp
    ..((........))..((((((((((..(((..(((((((((((.(((((.((((..(......)..)))).))))).)))))))))))..)))..)))).)))..))).   reads      mm        sample
    .................................cauuccaaggcuugccauuca........................................................   3          0         seq
    .................................cauuccaaggcuugccauucauc......................................................   1          0         seq
    ..................................auuccaaggcuugccauucauc......................................................   2          0         seq
    ....................................................................ugauuggcacgucuuggaau......................   4          0         seq
    ....................................................................ugauuggcacgucuuggaaug.....................   1          0         seq
    ....................................................................ugauuggcacgucuuggaauga....................   9          0         seq
    ....................................................................ugauuggcacgucuuggaaugaa...................   2          0         seq
    .....................................................................gauuggcacgucuuggaau......................   1          0         seq
    ......................................................................auuggcacgucuuggaau......................   5          0         seq
    ......................................................................auuggcacgucuuggaaug.....................   6          0         seq
    ......................................................................auuggcacgucuuggaauga....................   57         0         seq
    ......................................................................auuggcacgucuuggaaugaa...................   22         0         seq
    ......................................................................auuggcacgucuuggaaugaag..................   2          0         seq
```
